# Healthy older adults' preferences for home care services

**DOI:** 10.3389/fpubh.2026.1967098

**Published:** 2026-10-16

**Authors:** Xiaoke Yang, Tao Chen, Junhua Ke, Hui Lin, Wenxi Fan

**Affiliations:** 1 School of Humanities, Fujian University of Technology, Fuzhou, China; 2 The Affiliated Rehabilitation Hospital of Fujian University of Traditional Chinese Medicine, Fuzhou, China; 3 Fujian Key Laboratory of Rehabilitation Technology, Fuzhou, China

**Keywords:** care services, choice experiment, healthy older adults, mixed logit model, willingness to pay

## Abstract

**Background:**

As the aging population in China continues to grow, understanding the preferences and willingness to pay (WTP) for home care services among healthy older adult citizens becomes increasingly important. Most existing research focuses on disabled or semi-disabled older adult individuals, leaving a gap in knowledge regarding the preferences of those who are healthy and capable of self-care.

**Objectives:**

This study aims to explore the WTP for various home care services among healthy older adult citizens in Xi'an, China, and to analyze how different socio-demographic factors influence these preferences.

**Methods:**

A discrete choice experiment (DCE) was conducted to elicit preferences for home care services among a sample of healthy older adult citizens. The experiment included four non-monetary attributes (healthcare services, safety services, household affairs, and emotional care) and one monetary attribute (payment). A mixed logit model was used to estimate individual-level WTP for these services, followed by a Tobit regression analysis to identify socio-demographic factors influencing WTP.

**Results:**

The results indicate a significant positive WTP for all care services, with home repair (162 Yuan/month), traditional Chinese medicine physiotherapy (125 Yuan/month), and social activities (103 Yuan/month), being the most valued services. On average, respondents were willing to pay 628 Yuan per month for all care services combined. Heterogeneity in WTP was observed, with higher WTP among women, those living alone, those with worse health status, those with employee medical insurance and those with less financial pressure.

**Conclusions:**

The findings suggest that healthy older adult citizens in China have diverse needs for home care services, highlighting the importance of tailoring these services to specific subgroups. By addressing the varying preferences and WTP among the older adult population, service providers and policymakers can better meet the needs of this growing demographic, ultimately improving their quality of life. Further research is needed to generalize these findings to other regions and to explore the longitudinal changes in preferences as individuals age.

## 1 Introduction

The aging of the population has become a salient phenomenon in recent decades. According to the National Bureau of Statistics, the number of Chinese age 60 and older reached 296.97 million at the end of 2023, accounting for 21.1% of the total population (1). It is predicted that China will become a super-aged society by 2030 (2), which highlights the urgency of addressing the multifaceted challenges associated with this demographic transition.

Traditionally, older adults in China prefer to age at home due to the traditional value of filial piety rooted in Confucian philosophy (3). In response, the Chinese government has promoted the “9073” model for older adult care, where 90% of older adults are cared for by their families, 7% receive community care, and only 3% are cared for in professional institutions (4). While home-based care remains the primary option, there is growing evidence that the traditional family support system is weakening (5–7). Middle-aged women, who are typically the primary home caregivers (8), facing increasing challenges in providing adequate older adult care due to the conflict between extending life expectancy and their participation in the workforce (9). Furthermore, home caregivers are at a higher risk of experiencing psychological and physical health issues (10–12). As the demand for care services continues to grow, formal support becomes necessary while elderly people's demands for care services have been steadily on the increase (13). Additionally, the growing older adult population exacerbates the shortage of older care resources in China (14). A limited number of older adult care institutions, along with the quality of older adult care workers and the services provided, lag behind the rising demand of older adults population (7). Therefore, developing the older adult care service industry is crucial to effectively address this growing demand. Understanding the specific needs and preferences of older adults regarding care services is key to the industry's development.

Most existing research on older adult care has focused on disabled and semi-disabled individuals, emphasizing the importance of medical care services, including access to medical facilities and professional staff (15, 16). However, the health and functional status of older adults are diverse (17), and care service demands vary significantly among those with different living conditions (18). Recently, a report demonstrates that the average self-care ability of older adults in China has improved, and the disability rate has shown a downward trend (4). This raises questions about what aspects of care services are highly valued by older adults who possess the self-care abilities, referred to as “healthy older adults.”

Emerging literature emphasizes that the needs of older adult care extend beyond medical attention (5, 17). The 2022 Older adult Care Consumption Survey Project Research Report found that older adults have a higher demand for home-based care that focuses on daily needs, such as housekeeping and social interaction (19). Additionally, emotional support, companionship, and assistance with activities of daily living (ADLs) are also desired by healthy older adult individuals (5, 17). These findings suggest that a shift toward a more comprehensive care model is essential for improving the older adult care industry (20). Developed countries such as the United States, Canada, and Japan have implemented advanced older adult care models, providing four major services: protective, preventive, supportive, and social services. Protective services offer medical support to maintain overall health; preventive services enhance residential safety; supportive services assist with ADLs and instrumental tasks; and social services promote social engagement and psychological health (21). However, since a comprehensive older adult care model is still developing in China, there is insufficient evidence regarding the preferences of Chinese older adult care for such care packages. Therefore, further research on home care service options for older adults is needed.

To address these issues, it is crucial for the older adult care industry to integrate older adult preferences into its service design. Stated preference elicitation methods, such as discrete choice experiments (DCEs), can effectively capture these preferences (22, 23). The diversity of older adult care needs, coupled with varying preferences, necessitates a deep understanding that can be achieved through DCE research (24). This study aims to provide new insights into the preferences and demands of Chinese healthy older adult citizens for home care services by eliciting and quantifying their preferences for various attributes of these services using a DCE. We also assess the heterogeneity in willingness to pay (WTP) across different home care service attributes. The findings of this study will provide valuable evidence for designing and improving home care services tailored to the needs of the healthy older adults in China, ultimately enhancing their quality of life.

## 2 Materials and methods

DCEs have become a prominent method for eliciting preferences and willingness to pay (WTP) across various health and care contexts, including patient care, caregiver support, and general population needs (25, 26). This methodology is particularly effective for understanding the relative importance of specific attributes associated with hypothetical products or services (27). DCEs have been successfully applied to a range of older adult care settings, including home care services in Australia (25), long-term care services in Germany (23), nursing homes in China (28), various service aspects in the Netherlands (29), and home care services for older people with dementia in Italy (30). In this study, we employed a DCE approach to investigate the preferences of healthy older adult citizens regarding home care services in Xi'an, China. The DCE methodology involves several stages: identifying relevant attributes and their levels, designing the experimental framework and choice sets, developing the survey instrument, conducting the survey, and analyzing the data (31, 32).

### 2.1 Attributes and levels

To determine the attributes and levels for this study, a three-step approach was employed. First, an initial set of attributes was identified through a comprehensive literature review (see references in Table 1) and focus group discussions (see Additional file 1). These discussions involved a panel of six experts from relevant fields, including two gerontology experts, two public health experts, and two older adult care experts, together with 10 healthy older adult citizens, ensuring that the selected attributes accurately reflected the needs and preferences of this population for home care services. Second, a pilot test was conducted with a small sample of 35 healthy older adult participants to evaluate the clarity and relevance of the chosen attributes and levels. Feedback from the pilot test was used to refine the attribute descriptions to ensure they were easily understood. Finally, based on insights gathered from the pilot test and further expert consultations, the attributes and levels were finalized. The four non-monetary attributes were defined as broad service domains. Each domain included a “none” option and two specific service types identified through the literature review, focus group discussions, and pilot testing. These service types were treated as categorical levels in the analysis. The final design included four non-monetary attributes, namely medical care, safety care, household affairs management, and emotional care, each with three levels, together with one monetary attribute representing varying monthly payments. This systematic approach ensured that the discrete choice experiment was well-designed to capture the preferences of healthy older adult citizens regarding home care services. Table 1 provides a summary of the attributes and their respective levels for this study.

**Table 1:** Attributes and levels for eliciting preferences of older adult citizens.

Attributes	Description	Levels
Healthcare services (HS)	Basic monitoring involves regular measurements of vital signs such as heart rate, blood pressure, and blood sugar (39, 46), while physiotherapy in Traditional Chinese Medicine (TCM) include electronic massage, moxibustion treatment, foot reflexology for older adults (47).	**(1) None**^**a**^; (2) Basic monitoring; (3) Physiotherapy in TCM.
Safety services (SS)	The one-button call system allows older adults to call for help with a single touch in case of emergencies, while home safety assessments evaluate potential hazards in the home environment and suggest improvements every month (40–43).	**(1) None**^**a**^; (2) One-button call system; (3) Home safety assessment.
Household affairs management (HAM)	Home repair services cover essential maintenance tasks, such as repairs for water, electricity, and other essential utilities. Bill payment services assistance with paying bills and managing other financial affairs.	**(1) None**^**a**^; (2) Home repair services; (3) Bill payment services
Emotional care services (ECS)	Weekly phone calls involve regular conversations to provide emotional support, while bi-weekly social activities include organized events to enhance social interaction and reduce feelings of loneliness (38, 44).	**(1) None**^**a**^; (2) Phone calls; (3) Social activities.
Monthly payment (MP)	This monetary attribute represents the cost of the home care services and is expressed in terms of monthly payments in yuan ^b^.	**0**^a^; 50, 100, 150, 200.

^a^The status quo of each attribute, and have been used for experiment design except the zero payment; ^b^Chinese monetary unit, 7.0467 yuan = 1 USD in 2023 on average.

### 2.2 Experimental design and questionnaire development

In designing the DCE, careful attention was given to constructing the choice sets to capture a broad range of preferences among healthy older adult citizens regarding home care services. Each choice set presented respondents with three alternatives: two hypothetical scenarios featuring different combinations of care service attributes and a “Neither” option. Including the “Neither” option allowed participants to indicate if neither of the provided scenarios met their preferences, thereby enhancing the robustness of the findings by ensuring that the recorded choices reflected genuine preferences (31).

To generate the choice sets, we utilized Ngene software with an efficient design approach, specifically the D-optimality criterion with Multinomial Logit priors (eff = (mnl, d)). This method optimizes the statistical efficiency of the design by minimizing the variance of the estimated parameters, thereby improving the precision of the results (33). The priors were derived from a pilot study involving 86 respondents, providing initial estimates for the model. A total of 36 choice sets were created and divided into 12 blocks, with each respondent randomly assigned to one block, thus evaluating three choice sets. This blocked design helped reduce the cognitive burden on respondents while allowing for a thorough examination of preferences across different combinations of attributes.

To facilitate respondents' understanding of the choice tasks, each service level was presented using both text and corresponding illustrations. The visual presentation has been used in other literature intended to improve comprehension and reduce the cognitive burden of choice tasks (34). An example choice set is shown in Figure 1.

**Figure 1:**
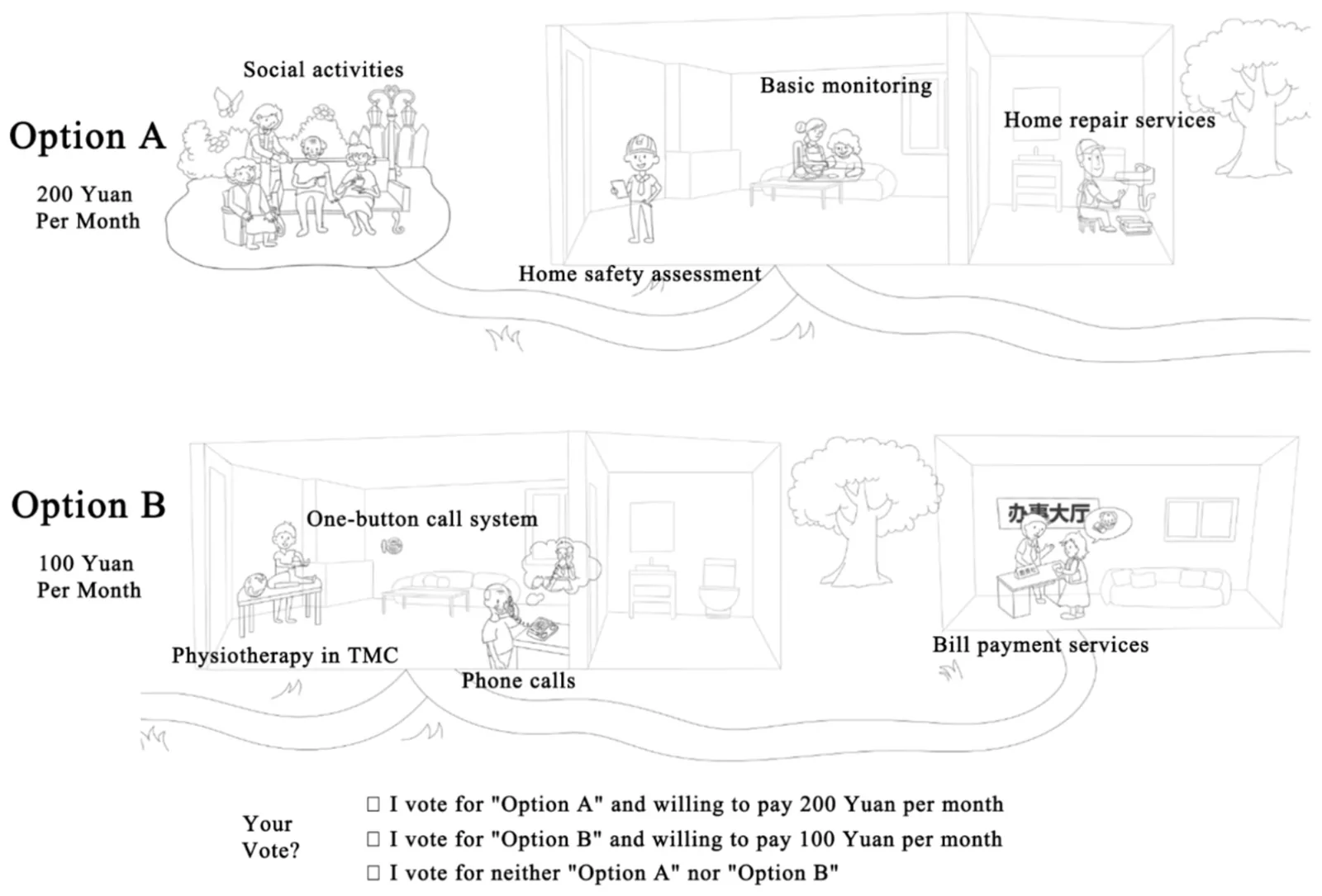
Example of the choice set for eliciting older adult citizens' preferences.

In addition to the DCE, the questionnaire included several sections to gather comprehensive information about the respondents. Questions were designed to capture attitudes toward home care services, ensuring that respondents felt comfortable with the interview process. Socioeconomic characteristics were also collected to identify potential heterogeneity in preferences during subsequent model analysis. To detect response biases, the questionnaire included specific items (31, 32). Respondents who chose the “Neither” option in all three scenarios were asked to indicate their reasons. If a respondent selected “I do not believe in this kind of service” as their reason for choosing zero payment, their responses were flagged as potential protest responses. Furthermore, questions aimed at detecting response biases included self-assessment items, where respondents were asked if they understood the choices presented, answered the choice tasks sincerely, and whether they would be willing to pay in a real-life situation. Interviewers also provided assessments, noting whether the respondents cooperated with the survey, understood the choices, and took sufficient time to answer the choice questions. This comprehensive approach ensured the collection of high-quality data, suitable for robust analysis and interpretation.

### 2.3 Survey administration

The data collection for this study was conducted through a face-to-face survey in Xi'an, China, from July 15th to August 7th, 2024. Xi'an, the capital of Shaanxi Province in Northwest China, has a significant aging population. According to the Seventh National Population Census, as of 2020, Xi'an had a resident population of approximately 12.95 million, with 2.0753 million people aged 60 and above, accounting for 16.02% of the total population. By 2025, the city's population is expected to reach 15 million, with the proportion of those aged 60 and above exceeding 3 million, or more than 20% of the total, classifying it as a moderately aging society (35). As the older adult population grows, Xi'an faces an increasing demand for home care services, alongside a relatively insufficient supply. Enhancing the quality of these services is crucial for improving the well-being of older adults and meeting their growing needs. In June 2023, the Xi'an Civil Affairs Bureau drafted the “Implementation Plan for Promoting the Construction of the Basic Older adult Care Service System,” which encourages public-private partnerships and professional service organizations to provide inclusive home care services, such as day care, recreational activities, health education, and home services based on the needs of older adults.

Given this context, we employed a multistage random sampling method to capture the preferences of healthy older adult citizens regarding home care services in Xi'an. The survey was conducted in six main districts of Xi'an: Xincheng, Beilin, Lianhu, Weiyang, Yanta, and Baqiao. Within each district, nine residential communities were selected, representing housing price percentiles from the 10th to the 90th based on an ordered list of average housing prices. In this study, we recruited healthy older adult respondents who were community dwelling older adults, able to care for themselves in daily life, and able to participate in the survey independently. The term “healthy older adults” was used to describe their general functional independence and did not imply the absence of chronic diseases or other health problems. Paper-pencil surveys, lasting approximately 10–20 minutes to complete, were administered in the public spaces of each community. From each community, 30 older adult individuals aged 60 and above were randomly chosen, resulting in a sample size of 1,620 participants.

To ensure consistency and reliability, the face-to-face surveys were conducted by 10 well-trained interviewers, each of whom underwent 1 days of training. Interviewers followed a standardized protocol, which included obtaining informed consent, explaining the survey's purpose and procedures, and assisting respondents only when necessary to ensure comprehension of the choice tasks. Participants were assured of confidentiality and informed that their participation was voluntary, with the option to withdraw at any time without consequences.

### 2.4 Model Specification

We estimated the preferences of healthy older adult citizens using a mixed logit model (36). The utility *U*_*ijt*_ that respondent *i* derives from choosing alternative *j* in choice situation t is specified as:


$$\begin{array}{l} U_{ijt}=\;\beta_{T}^{i}X_{ijt}+\;\varepsilon_{ijt=\;}\alpha_{i}^{T}D_{jjt}+\gamma_{i}\;p_{ijt}\;+\;\varepsilon_{ijt} \end{array}$$ (1)

In the Equation 1, *X*_*ijt*_ is a vector of attributes for each alternative. Dummy coding was applied to all the non-monetary attributes (*D*__*ijt*_)_ and the monthly payment per person (*p*_*ijt*_) was treated as continuous numeric variable. The vector β_*i*_ represents individual-specific random coefficients, effectively capturing the diverse preferences among the older adult population for different attributes of home care services. The term ε_*ijt*_ denotes the error term, which follows an independent extreme value distribution.

Based on the estimated utility function, the respondent's WTP for a specific home care scenario can be derived from the Equation 2:


$$\begin{array}{l} WTP_{i}=\left(\alpha_{j}^{T}\;D_{ij}^{l}\right)/\gamma_{j} \end{array}$$ (2)

where the value of $D_{ij}^{1}$ is the attributes' non-zero levels in the specified s scenario. With the estimation of individual level parameters, we further explore the heterogeneity of WTP using the Equation 3:


$$\begin{array}{l} WTP_{i}=\rho^{T}\;Z_{j}+\;e_{j} \end{array}$$ (3)

where *Z*_*i*_ represents the individual characteristics that may affect *WTP*, ρ measures the impact of these characteristics, and *e*_*i*_ is the standard normal distributed error term.

## 3 Results

### 3.1 The Sample

The face-to-face survey conducted in Xi'an resulted in the collection of 1,283 valid questionnaires out of 1,620 participants, yielding a valid rate of 79.2%. The remaining 337 questionnaires were excluded due to various response biases and quality concerns. Specifically, 71 responses were identified as protest zeros, additional 186 responses were flagged for potential biases based on self-assessment questions, primarily due to respondents' uncertainty about their willingness to pay in a real-life context, and another 80 responses were excluded based on interviewer assessments, which highlighted issues such as lack of cooperation or insufficient understanding of the choice tasks. This rigorous screening process ensured the validity and reliability of the data, providing a robust foundation for subsequent analysis. Table 2 presents the descriptive statistics of the final sample of 1,283 respondents.

**Table 2:** Descriptive statistics of the surveyed older adults' characteristics (*N* = 1,283).

Variables	Description	Statistics	
Gender	Female = **0**	Ratio	0.652
	Male = **1**	Ratio	0.348
Age	Age of the respondents, continues variable	Mean	69.401
		Std. dev.	4.964
	[60, 69]	Ratio	0.575
	[70, 79]	Ratio	0.394
	Above 79	Ratio	0.034
*Edu*	Education level of the respondents with four categories	Mean	1.834
		Std. dev.	0.749
	Primary school or under = **1**	Ratio	0.330
	Junior school = **2**	Ratio	0.554
	Senior school = **3**	Ratio	0.069
	College degree or above = **4**	Ratio	0.048
Health	Self-reported health status of respondents measured on a five-point scale	Mean	2.645
		Std. dev.	1.018
	Significant health problems limit daily activities = **1**	Ratio	0.126
	Some problems occasionally limit my activities = **2**	Ratio	0.336
	Generally fine, but have some ongoing concerns = **3**	Ratio	0.348
	In good health with only minor health issues = **4**	Ratio	0.146
	Very healthy and have no health problems = **5**	Ratio	0.044
Pressure	Current financial situation in terms of economic pressure in three-point Likert scales	Mean	2.033
		Std. dev.	0.569
	Finances can comfortably meet my needs = **1**	Ratio	0.146
	Generally manage my expenses = **2**	Ratio	0.676
	Struggle to cover my expenses = **3**	Ratio	0.179
Alone	Living with family members = **0**	Ratio	0.857
	Living alone without family members = **1**	Ratio	0.143
Insurance	Endowment insurance for urban workers = **1**	Ratio	0.327
	Other insurance or no insurance = **0**	Ratio	0.673
Vote	“Option A” has been chosen	Ratio	0.436
	“Option B” has been chosen	Ratio	0.406
	“Neither” has been chosen	Ratio	0.158

Our survey targeted older adult citizens aged 60 and above, with an average age of 69.4 years. The sample included a higher proportion of females (65.2%) compared to males. Educational backgrounds varied, with most participants having a middle school education, followed by those with primary, high school, and post-secondary education. Most respondents resided with their spouse or children, while approximately 15% reported significant economic pressure. Although the respondents were able to participate in the survey independently, 46.2% reported varying degrees of health-related limitations, indicating heterogeneity in self-reported health status within the sample. Additionally, most participants were covered by endowment insurance for urban workers. After excluding protest responses, 15.8% of the choice tasks were chosen with the zero-payment option. These sample characteristics align with data from the Xi'an Statistical Bureau (35) and other provincial capital city (14), reflecting the representativeness of our sample.

### 3.2 Mixed Logit Model Estimation

The Mixed Logit model was used to analyze respondents' preferences. The following specifications were applied: (1) An Alternative Specific Constant (ASC) was introduced, taking the value of 1 for the “Neither” option and 0 otherwise, representing the baseline utility in the absence of home care services. (2) Eight non-monetary binary variables were dummy-coded: basic monitoring (monitoring), physiotherapy in traditional Chinese medicine (physiotherapy), one-button call system (system), home safety assessment (assessment), home repair services (repair), bill payment services (bill), phone calls (phone), and social activities (social). (3) Since the marginal utility of expenditure is always negative, a parameter for willingness to pay was set with a negative sign, and γi was assumed to follow a log-normal distribution. (4) For the eight non-monetary dummy variables, the parameter distributions were assumed to be normal. Estimation results were obtained using Stata software with 2,000 Halton draws, as shown in Table 3.

**Table 3:** Results of the mixed logit model.

Variable	Mean		Std. dev.	
	*coef*.	*std. err*.	*coef*.	*std. err*.
ASC	−0.991^***^	0.297	
Monitoring	1.039^***^	0.137	1.446^***^	0.255
Physiotherapy	3.298^***^	0.296	2.122^***^	0.376
System	0.602^***^	0.141	1.127^**^	0.44
Assessment	1.226^***^	0.146	0.366	0.642
Repair	4.288^***^	0.409	0.542	0.534
Bill	0.701^***^	0.157	3.533^***^	0.321
Phone	1.687^***^	0.182	2.735^***^	0.297
Social	2.71^***^	0.233	1.129^***^	0.338
*p* (logged)	−3.335^***^	0.106	0.772^***^	0.044
Log likelihood (LL)	−2923.4811^***^			
Pseudo ^*R*2^	0.255			
Number of obs	3849 (i.e., 1283^*^3)			

^*^, ^**^ and ^***^ indicate the significant level of 10%, 5% and 1% respectively.

Based on the overall model fit, the log-likelihood test is significant at the 1% level, and the McFadden Pseudo R^2^ is greater than 0.2, indicating that the model is statistically significant as a whole. The marginal utility of the fixed parameter ASC is significantly negative (α=-0.991, *p* < 0.01), suggesting that older adult citizens have an inherent preference for improving the current state of home care services. The mean parameter estimates for the eight non-monetary dummy variables are all significantly positive, indicating that, on average, all care services significantly increase utility levels, which aligns with theoretical expectations. Given that the coefficient γi follows a log-normal distribution, we can compute the point estimates for the mean, and variance of γi as−0.048 and 0.043, respectively.

In terms of the standard deviations of the random parameters, the estimates for preferences toward home repair services (repair, α = 0.542, *p* = 0.310) and home safety assessment (safety, α = 0.366, *p* = 0.569) are not statistically significant. This suggests consistency among respondents for these attributes, indicating no substantial variation in preferences. However, the significant standard deviations for other attributes' levels, such as bill payment services (bill), one-button call system (system), phone calls (phone) and basic monitoring (monitoring) indicate substantial variation in preferences. Specifically, 42%, 30%, 27%, and 24% of respondents perceive these attributes as having a negative marginal impact on their utility, respectively. For all other significant attributes' levels, the proportion of respondents for whom the marginal impact on utility is negative is below 10%. Overall, these results suggest that a further heterogeneity analysis of preferences is necessary.

### 3.3 WTP Estimation

To calculate WTP for each of the eight non-monetary normally distributed coefficients and a log-normally distributed payment coefficient, the Krinsky and Robb method (37) has been used. This method allows for proper confidence interval estimation by simulating the distributions of the parameters involved. Table 4 reports the simulated WTP statistics for the non-monetary coefficients, with the compensating surplus (CS) aggregated based on improvements in all eight non-monetary variables.

**Table 4:** Results of the WTP estimations from Krinsky and Robb method.

*Attributes*	*Levels*	*Mean*	*std. dev*.	*p1*	*p50*	*p99*
	ASC	−37.564	11.674	−67.590	−36.787	−11.764
Healthcare services	Monitoring	39.487	6.581	25.726	39.219	55.319
	Physiotherapy	125.175	11.341	102.312	124.315	154.225
Safety services	System	22.518	4.947	11.338	22.444	34.853
	Assessment	46.725	6.703	32.698	46.076	63.933
Household affairs	Repair	162.053	12.903	135.559	161.434	196.837
	Bill	26.991	8.457	8.650	26.635	49.352
Emotional care	Phone	64.588	9.895	44.499	63.852	90.064
	Social	102.825	10.352	80.827	101.932	130.878
CS		627.927	53.662	517.941	623.075	776.732

The estimation results indicate that the monthly WTP for all care services is significantly positive within the 99% two-sided confidence interval. The ranking of WTP for care services, from highest to lowest, is as follows: home repair services (repair, WTP=162 Yuan/month), physiotherapy in traditional Chinese medicine (physiotherapy, WTP=125 Yuan/month), social activities (social, WTP=103 Yuan/month), phone calls (phone, WTP=65 Yuan/month), home safety assessment (assessment, WTP=47 Yuan/month), basic monitoring (monitoring, WTP=39 Yuan/month), bill payment services (bill, WTP=27 Yuan/month), and one-button call system (system, WTP=23 Yuan/month). The total compensating surplus suggests that respondents are, on average, willing to pay 628 yuan per month for all care services.

### 3.4 Preference Heterogeneity Analysis

Individual-level WTP was calculated using the ‘mixlbeta‘ command in Stata. Given that individuals with negative WTP would realistically opt not to pay for these services, we set any calculated negative WTP values to zero. Subsequently, we used linear regression to analyze the factors influencing WTP, with the results presented in Figure 2.

**Figure 2:**
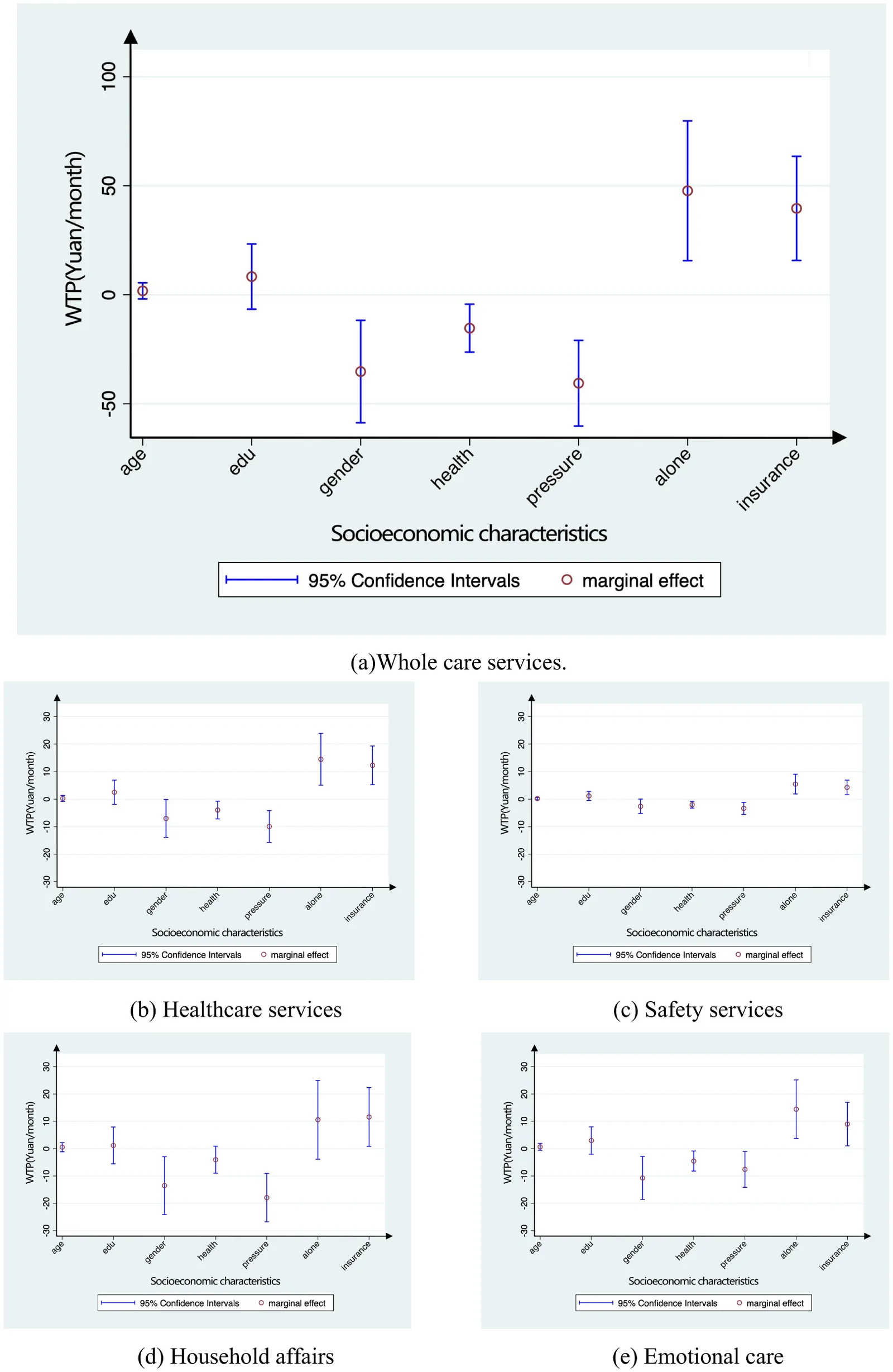
Marginal effects of socioeconomic characteristics on WTP: **(a)** whole care services; **(b)** healthcare services; **(c)** safety services; **(d)** household affairs; and **(e)** emotional care.

Several socioeconomic characteristics significantly influenced WTP for home care services, including gender, health status, financial pressure, living arrangements, and insurance type. The results indicate that male respondents generally have a lower WTP for care services compared to female respondents (β = −36.396, *p* < 0.01), primarily due to their lower WTP for household affairs and emotional care services. Older adult citizens in better health (β = −16.620, *p* < 0.01) and those experiencing greater financial pressure (β = −40.200, *p* < 0.01) also show a lower overall WTP for care services, particularly for healthcare services and household affairs management. Conversely, older adult citizens who live alone exhibit a higher overall WTP for care services (β = 46.497, *p* < 0.01), especially for healthcare services and emotional care. Additionally, the type of medical insurance held by older adult citizens significantly affects their overall WTP (β = 43.865, *p* < 0.01), with those covered by “Endowment Insurance for Urban Workers” demonstrating a notably higher WTP than those with other types of insurance.

## 4 Discussion

This study investigates the WTP of healthy older adult citizens for various aspects of home care services, specifically focusing on healthcare services, safety services, household affairs, and emotional care. To the best of our knowledge, few studies have specifically examined the preferences of healthy older adult citizens for a broad range of home care services in China. Our findings extend previous research, which primarily concentrated on the preferences of disabled or semi-disabled older adult individuals for medical and nursing services. However, healthy older adult citizens constitute a larger proportion of the older adult population (about 85%) (35), and the results of this study indicate that all attributes included in the choice experiment are statistically significant, suggesting that respondents placed positive value on multiple dimensions of home care services.

As anticipated, respondents demonstrated a significantly positive WTP for all care services included in the DCE. The most valued service was home repair services (repair), an area that has not received sufficient attention in existing research. A potential reason for this highest WTP is the challenges older adult citizens face in managing modern home appliances, coupled with issues such as non-transparent repair information, exaggerated minor problems, substandard repairs, high costs, and poor service quality. According to the theory of information asymmetry in economics, outsourcing repair services at a fixed price can mitigate the high costs associated with non-transparent repair practices. Following home repair services, respondents showed the second-highest WTP for physiotherapy in TCM (physiotherapy). This preference may be due to a growing interest among healthy older adult citizens in wellness practices, shifting their focus from “disease treatment” to “disease prevention” and emphasizing health maintenance to reduce or avoid illness (5, 17). Additionally, respondents ranked their WTP for bi-weekly social activities (social) third. This finding suggests that the current social needs of older adult citizens are not being effectively met (38). A potential reason for this could be the lack of accessible and engaging social opportunities that cater to the interests and mobility of the older adult population, leading to a greater demand for organized social activities that foster community and connection among older adults.

For the remaining five services, the average WTP was below 100 yuan. Notably, the lowest WTP was for the one-button call system (system), which may partly reflect the widespread availability of smartphones and smart voice assistants, as these technologies may provide functions similar to those of the one-button call system. The WTP for bill payment services (bill) was the second lowest, with the highest variability in preferences among respondents. On one hand, healthy older adult citizens with children may receive assistance from family members in paying utility bills online through mobile applications, which could reduce their need for this service. In addition, in urban areas with well-developed public infrastructure, bill payments can be easily made at nearby smart service terminals. Our findings also align with the literature (8, 39), indicating that older adults have a moderate WTP for basic health monitoring (monitoring), such as measuring vital signs like heart rate, blood pressure, and blood sugar. While the necessity of aging-friendly home modifications (assessment) is frequently discussed in the literature (40–43), our findings provide evidence that respondents also placed a positive monetary value on home safety assessment services. Specifically, healthy older adult citizens in our sample were willing to pay approximately 47 yuan per month for home safety assessments, suggesting potential demand for such services among relatively healthy older adults. Lastly, consistent with previous studies (38, 44), our findings suggest that respondents also valued regular phone calls (phone) for emotional support. Recent data suggest that approximately 85% of adults aged 60 years and older in Xi'an's main urban areas can be classified as healthy older adult citizens (17, 37). Based on the Seventh National Population Census, the total older adult population in these areas is approximately 0.81 million, corresponding to an estimated healthy older adult population of around 0.69 million. Applying the estimated average WTP from this study to this population yields an annual aggregate WTP of approximately 5.19 billion yuan. This amount is equivalent to about 0.6% of the Gross Domestic Product (GDP) of Xi'an's main urban areas in 2024 and may indicate substantial potential demand for home care services among healthy older adult citizens.

This study also identifies heterogeneity in WTP through an analysis of the characteristics influencing individual WTP. Higher WTP was observed among females, those living alone, and those with less financial pressure. These findings align with previous studies conducted in Hubei Province (24), Lanzhou City (14), and Chongqing City (45, 48). Additionally, our study found that healthy older adult citizens with employee medical insurance exhibit a higher WTP. A possible explanation is that these respondents may have greater financial capacity to afford home care services. This is consistent with our finding that lower financial pressure was associated with higher WTP.

Lastly, this study identifies the factors influencing WTP for healthcare services, safety services, household affairs, and emotional care. These findings highlight how individual characteristics affect the structure of WTP. Specifically, healthy older adult citizens living alone showed a higher WTP for emotional care and household affairs, while respondents with poorer self-reported health showed a higher WTP for healthcare services. Female respondents also exhibited a higher WTP for safety services. Taken together, these patterns suggest that demographic, living-arrangement, and health characteristics may be relevant when designing differentiated home care service packages for healthy older adult populations.

## 5 Conclusions and limitations

(1) Conclusions

This study shows that healthy older adult citizens value multiple dimensions of home care services, including healthcare, safety, household affairs, and emotional care. The findings suggest that the needs of functionally independent older adults extend beyond traditional medical support and include practical household assistance, preventive care, and opportunities for social engagement.

The substantial variation in WTP across service types indicates that home care services should not be designed as a uniform package. Instead, service provision may benefit from prioritizing the types of support that healthy older adult citizens value most and from offering flexible combinations of services. The observed heterogeneity in WTP across gender, health status, financial pressure, living arrangement, and insurance type further suggests that different groups of older adults may require different service configurations.

In general, these findings support a broader approach to home based older adult care that includes healthy and functionally independent older adults alongside populations with greater care needs. Incorporating preference information into service planning may help improve the allocation of care resources and support the development of more differentiated and responsive home care services.

(2) Limitations

However, this study has several limitations that should be considered. First, DCE may not fully capture the complexity of the healthy older adult citizens' preferences for home care services. Future studies could expand the range of attributes to include other relevant factors such as cultural influences, regional differences in care expectations. Second, the sample of this study is restricted to healthy older adult citizens from Xi'an, China, future research should aim to include a more diverse sample from multiple regions across China, accounting for variations in socioeconomic status, access to care, and cultural norms. Finally, the cross-sectional design of this study captures preferences at a single point in time, without accounting for how these preferences might change as individuals age or as their health conditions evolve. Future research could address these issues by adopting a longitudinal design to track changes in preferences over time. By recognizing these limitations, this study contributes to the understanding of the home care service needs of healthy older adult citizens, and provides a foundation for more comprehensive research that can offer a deeper understanding of the healthy older adult citizens' preferences for home care services, ultimately aiding in the development of more tailored and effective care options to enhance their quality of life.

## Data Availability

The datasets presented in this study can be found in online repositories. The names of the repository/repositories and accession number(s) can be found in the article/Supplementary material.
